# Mixed-methods evaluation of the implementation of IOTA-ADNEX ultrasound triage in NHS secondary care ovarian diagnostic one-stop clinics

**DOI:** 10.1136/bmjoq-2025-003909

**Published:** 2026-04-20

**Authors:** Vivian Do, Helen Crisp, Carole Cummins, Sarada Kannangara, Grisham Smotra, Becky Tarbuck, Omiete Duke, Aamena Salar, Nina Jhita, Vincent Sai, Naresh Rati, Sudha Sundar

**Affiliations:** 1 Department fo Cancer and Genomics, University of Birmingham College of Medical and Dental Sciences, Birmingham, UK; 2 Pan Birmingham Gynaecological Cancer Centre, Sandwell and West Birmingham Hospitals NHS Trust, Birmingham, UK; 3 Crisp Quality Improvement Consultancy Limited, London, UK; 4 Applied Health Sciences, University of Birmingham, Birmingham, UK; 5 Walsall Healthcare NHS Trust, Walsall, UK; 6 Modality Partnership, Birmingham, UK

**Keywords:** Obstetrics and gynecology, Implementation science, Diagnostic Imaging

## Abstract

**Objectives:**

Robust evidence supports International Ovarian Tumour Analysis (IOTA)-Assessment of Different Neoplasias in the Adnexa (ADNEX) ultrasound triage at 10% threshold for ovarian cancer (OC) diagnosis to identify women for referral to tertiary gynaecological cancer centres for further management. The IOTA-ADNEX risk prediction model has superior sensitivity compared with the current standard of care triage, Risk of Malignancy Index (RMI-1), yet NHS adoption is limited. In our survey of British Gynaecological Cancer Society clinicians, only 30% (24/79) currently follow an IOTA model, despite 80% (63/79) supporting implementation. We evaluated IOTA-ADNEX implementation within two NHS one-stop clinics (OSC) for suspected OC, examining clinical outcomes alongside implementation barriers and facilitators.

**Methods:**

Mixed-methods study conducted across two UK NHS hospitals between June 2023 and June 2025. Implementation outcomes were surgical intervention rates comparing IOTA-ADNEX-guided and retrospectively calculated RMI-based management using National Institute for Health and Care Excellence/Royal College of Obstetricians and Gynaecologists thresholds and patient process metrics. 11 qualitative semi-structured interviews were conducted with NHS staff involved in OSC implementation and thematic analysis performed.

**Results:**

Of 334 patients, 42% (139) underwent same-day discharge. Using IOTA-ADNEX at a 10% threshold, 10% (32/334) of patients underwent surgery under the general gynaecology and cancer unit team. In comparison, 30% (94/334) would have undergone surgery under the same teams if RMI-based triage had been used. Five themes identified from qualitative analysis: organisational infrastructure, clinical decision-making, communication and pathway definition, professional collaboration and training support, and patient experience. Key facilitators included dedicated clinical leadership, timely decision-making capabilities and quality assurance sessions. Barriers included lack of standardised post-clinic pathways and insufficient staff communication about pathway changes.

**Conclusions:**

IOTA-ADNEX implementation in OSC offers high same-day discharge rates and reduction in surgical rates compared with RMI triage. To ensure success, implementation should be supported by adequate infrastructure, training and clear pathways. It requires leadership, comprehensive staff training and robust communication strategies. These findings provide practical guidance for healthcare systems for wider implementation of IOTA-ADNEX.

**WHAT IS ALREADY KNOWN ON THIS TOPIC**

- The International Ovarian Tumour Analysis (IOTA)-Assessment of Different Neoplasias in the Adnexa (ADNEX) ultrasound risk-assessment model to triage adnexal masses has demonstrated superior diagnostic accuracy even when applied by certified non-expert sonographers over Risk of Malignancy Index (RMI), the standard tool in UK ovarian cancer pathways, but implementation within UK NHS pathways remains limited.

**WHAT THIS STUDY ADDS**

- This mixed-method evaluation shows IOTA-ADNEX ultrasound triage can be successfully implemented in NHS one-stop clinics, with the potential to reduce benign surgeries compared with RMI and enable high same-day discharge rates without missing invasive cancers. Findings on key facilitators and barriers highlight the need for infrastructure, training and pathway clarity when implementing this new diagnostic pathway and can potentially inform wider NHS adoption of IOTA-ADNEX under similar settings.

**HOW THIS STUDY MIGHT AFFECT RESEARCH, PRACTICE OR POLICY**

- Findings from this mixed-method evaluation can support broader NHS implementation of IOTA-ADNEX, further evaluation studies across diverse hospital settings would be helpful to strengthen the evidence base.

## Introduction

 Ovarian cancer (OC) represents a serious health challenge and remains the sixth most common cancer in the UK.^1^ Recent UK statistics are alarming: approximately 30% of women die within their first year after diagnosis and 70% present with advanced-stage diseases.^2^ Late-stage presentation dramatically impacts survival chances, where 5 year survival is only 13% (stage IV), compared with over 90% for stage 1.^1^ Diagnosis is often delayed due to vague symptoms including bloating and abdominal pain which can be attributed to less serious conditions. Efforts have been made to enhance diagnostic accuracy, particularly through robust triaging of adnexal (ovarian) masses, which ensures timely diagnosis and referral to the appropriate care setting. UK OC care is delivered through the NHS using a hub-and-spoke model. Local general hospitals (‘spoke’ sites) conduct initial assessments and manage lower-risk cases via the gynaecological cancer unit team. Patients with suspected or high-risk OC are referred to ‘hub’ centres, where specialised multidisciplinary teams (MDTs) including gynaecological oncologists are available to offer advanced surgery, cancer treatments, comprehensive perioperative and psychosocial support.^3^,^5^ Centralising these cases to specialised cancer centres improves patient outcomes, with 10% lower mortality compared with management in non-specialist settings (general hospital).^3^ This underscores the importance of accurate triaging to ensure that the right patients reach the right expertise at the right time.

The UK’s National Institute for Health and Care Excellence (NICE) recommends a stepwise referral pathway for suspected OC (online supplemental figure 1).^6^ Primary care physicians request a CA125 blood test (protein which can be raised in OC) for symptomatic women, followed by a pelvic ultrasound if raised. Abnormal scans prompt urgent hospital referral for patients to receive a definitive diagnosis within 28 days under NHS Faster Diagnosis Standards.^7^ In practice, referral patterns often deviate from this pathway, with patients referred with symptoms alone or with partial test results. Hospital doctors calculate the Risk of Malignancy Index (RMI), a score combining ultrasound findings, CA125 levels and menopausal status, to estimate the cancer risk.^6^ RMI scores <25 indicate low risk (<3% cancer probability), 25–250 moderate risk (20% cancer probability) and >250 high risk (70% cancer probability).^8^ High-risk patients (RMI ≥250) are referred to cancer centres for open surgery by gynae-oncologists, while lower risk patients (RMI <250) receive monitoring or minimally invasive surgery by local gynaecology or cancer unit teams. NICE recommends a threshold of 250, while the Royal College of Obstetricians and Gynaecologists (RCOG) applies 200 for postmenopausal women.^6 8^ Both offer comparable diagnostic performance, with sensitivities of 70% and 78% and specificities of 90% and 80%, respectively.^8^

Compared with RMI, the IOTA (International Ovarian Tumour Analysis)-ADNEX (Assessment of Different Neoplasias in the Adnexa) model offers a superior diagnostic accuracy (Area Under the Curve 0.93 vs 0.82) and misses less cancers with a higher sensitivity (96.1% vs 82.9%).^9^,^11^ It is a multiclass ultrasound risk-prediction model which differentiates between a non-cancerous and cancerous mass, and breaks down the cancer risk into borderline tumour, early-stage (stage I), advanced stage (stage II-IV) and secondary metastases (cancer has spread to the ovary from elsewhere). It remains accurate even when used by non-expert, IOTA-certified, quality-assured sonographers.^11 12^ Its adoption is supported by expert consensus and incorporated into British Gynaecology Cancer Society (BGCS) 2024 guidelines.^13 14^

During a single ultrasound appointment, ultrasound examiners apply the IOTA-ADNEX model using a two-step strategy to evaluate the adnexal masses. First examiners assess for specific ultrasound features termed ‘modified benign descriptors’ (BD) to identify masses likely to be benign with a cancer risk of <1%. If not applicable, the IOTA-ADNEX score is calculated.^15^ Validation in long-term studies shows complication rates <1% for conservatively managed benign masses over 2 years.^16^ Embedding this strategy into one-stop clinics (OSC), where patients receive consultation, ultrasound and management plan in a single visit, can streamline care, reduce unnecessary imaging and appointments. The OSC model has proven successful in other NHS gynaecological pathways, including endometrial cancer.^17 18^

Evidence on its implementation is limited; our systematic literature search identified one paper addressing current uptake and barriers of IOTA models in clinical practice.^19^ In our survey of BGCS clinicians, only 30% (24/79) currently follow an IOTA model, despite 80% (63/79) supporting implementation. Successful implementation requires more than clinical validation: it depends on infrastructure, workforce engagement, resources and consideration of patient experience. We explored the clinical impact, facilitators and barriers of implementation in a mixed-methods study.

## Methods

### Study design

This implementation service evaluation employed a convergent parallel mixed-method design, integrating quantitative and qualitative data to evaluate both clinical impact and implementation experience of the adoption of IOTA-ADNEX two-step strategy within NHS OSC (online supplemental figure 2). Quantitative and qualitative components were conducted concurrently, analysed independently and merged during interpretation to provide a comprehensive understanding of the intervention’s effectiveness and contextual feasibility.

### Quantitative component

**Figure 1:**
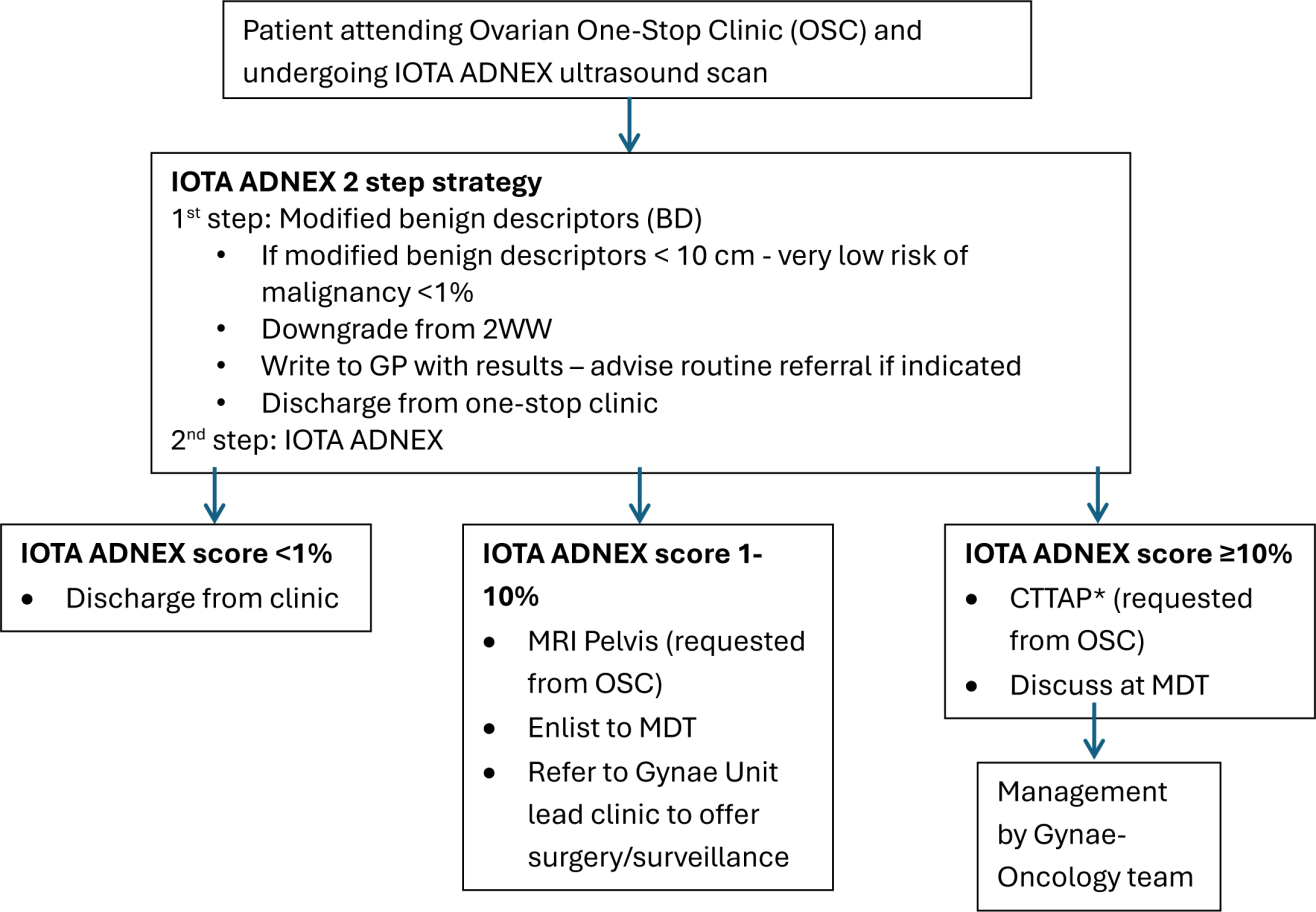
One-stop clinic flowchart used at SWBH (modified from 2021 ESGO guidance). *CTTAP, CT thorax, abdomen and pelvis. ADNEX, Assessment of Different Neoplasias in the Adnexa; IOTA, International Ovarian Tumour Analysis; MDT, multidisciplinary team.

A multicentre, prospective, observational cohort study evaluated symptomatic women attending weekly ovarian OSC at two NHS hospitals from June 2023 (Sandwell and West Birmingham, SWBH, a cancer centre) and October 2023 (Walsall Healthcare NHS Trust, a cancer unit) until June 2025. Clinics offered same-day gynaecological assessment and pelvic ultrasound (transabdominal and transvaginal) by IOTA-certified Level II examiners and reported using standardised IOTA terminology, providing a structured and standardised description of the masses.^20^ The IOTA-ADNEX two-step strategy was applied in the same appointment to triage masses, guiding clinical management based on the multi-panel expert consensus (2021) (figure 1). The IOTA-ADNEX calculator was made available through Professor Dirk Timmerman (now available as a medical device app through the Gynaia website).^21^ During early implementation, the IOTA Simple Rules (SR) model was used (n=24) due to temporary calculator unavailability. This model classifies adnexal masses as benign (‘B’ rules), malignant (‘M’ rules) or ‘Uncertain’ if both rules apply. Walsall used a single IOTA-trained gynaecologist for scanning and consultation, while SWBH employed IOTA-certified doctors working with trained sonographers.

Patient data were extracted from electronic health records including demographics, ultrasound findings, triage decisions, surgical outcomes and discharge timelines by NHS employed clinical research fellows. Data were stored and analysed securely on an Excel spreadsheet on NHS One Drive. Clinical decisions were based on IOTA-ADNEX triaging at 10% threshold applied in real-time, while RMI scores were retrospectively calculated using the formula: U (Ultrasound score) × M (menopausal status) × CA125 (IU/ml).^6^ We ascertained disease status by reviewing all subsequent healthcare presentations to both hospitals, using electronic record searches. MDT records from designated local gynaecological cancer centres were examined to identify any further relevant presentations.

*Inclusion criteria:* women referred via the urgent suspected OC pathway from primary or secondary care.

*Exclusion criteria:* pregnancy and patients who did not attend their appointment.

The primary outcome compared surgical intervention rates based on IOTA-ADNEX-guided decisions and retrospectively calculated, counterfactual RMI-based management using NICE/RCOG thresholds, that is, determining what the patient management would have been if based on RMI not IOTA-ADNEX. Secondary outcomes were process metrics: additional imaging requirements and discharge patterns (within 3 months of referral/same day). Patients not discharged during this period due to ongoing investigation and management were classified as ‘follow-up with surveillance’. Data were analysed using descriptive statistics calculated in Excel. Borderline ovarian tumours were classified within the ovarian tumour category for analysis purposes. In the UK, surgical management of suspected borderline tumours may be undertaken in either cancer unit or cancer centre settings.

### Qualitative component

This was a prospective, qualitative evaluation study, looking at the facilitators and barriers of IOTA-ADNEX implementation across the two NHS OSCs.

11 semi-structured interviews involving 14 purposively sampled NHS staff were conducted throughout the study period (SWBH: June 2023, Walsall: October 2023 until June 2025). Participants included gynaecologists, gynae-oncologists, sonographers, clinic and cancer service administrative staff. We obtained verbal and written consent via email from participants before interviews. Participants were informed about the evaluation’s purpose, voluntary nature of participation and confidentiality measures.

Virtual interviews (30–35 min) were conducted via Microsoft Teams/Zoom by an independent Quality Improvement consultant (HC) and a gynae-oncology research fellow based at SWBH (VD). Real-time transcripts were documented on Microsoft Word. Interviews were deemed to have achieved data saturation, as no new themes or insights emerged in the last few interviews. Inductive thematic analysis followed Braun and Clarke’s methodology, with independent coding by HC and VD.^22^ All interview data were anonymised with no identifiable information included in our reporting.

### Patient satisfaction survey

From February 2025, patients who attended the clinic were asked to complete a short, anonymised online patient satisfaction survey with open and closed questions (online supplemental figure 3). The survey could be accessed via a QR code shown on a poster within the clinic waiting area and was included in the patient clinic letter. It was developed with the support from the SWBH Patient Insight and Involvement team in conjunction with Patient Experience Analysts who helped provide clear, plain-language wording throughout.

## Results

**Figure 2:**
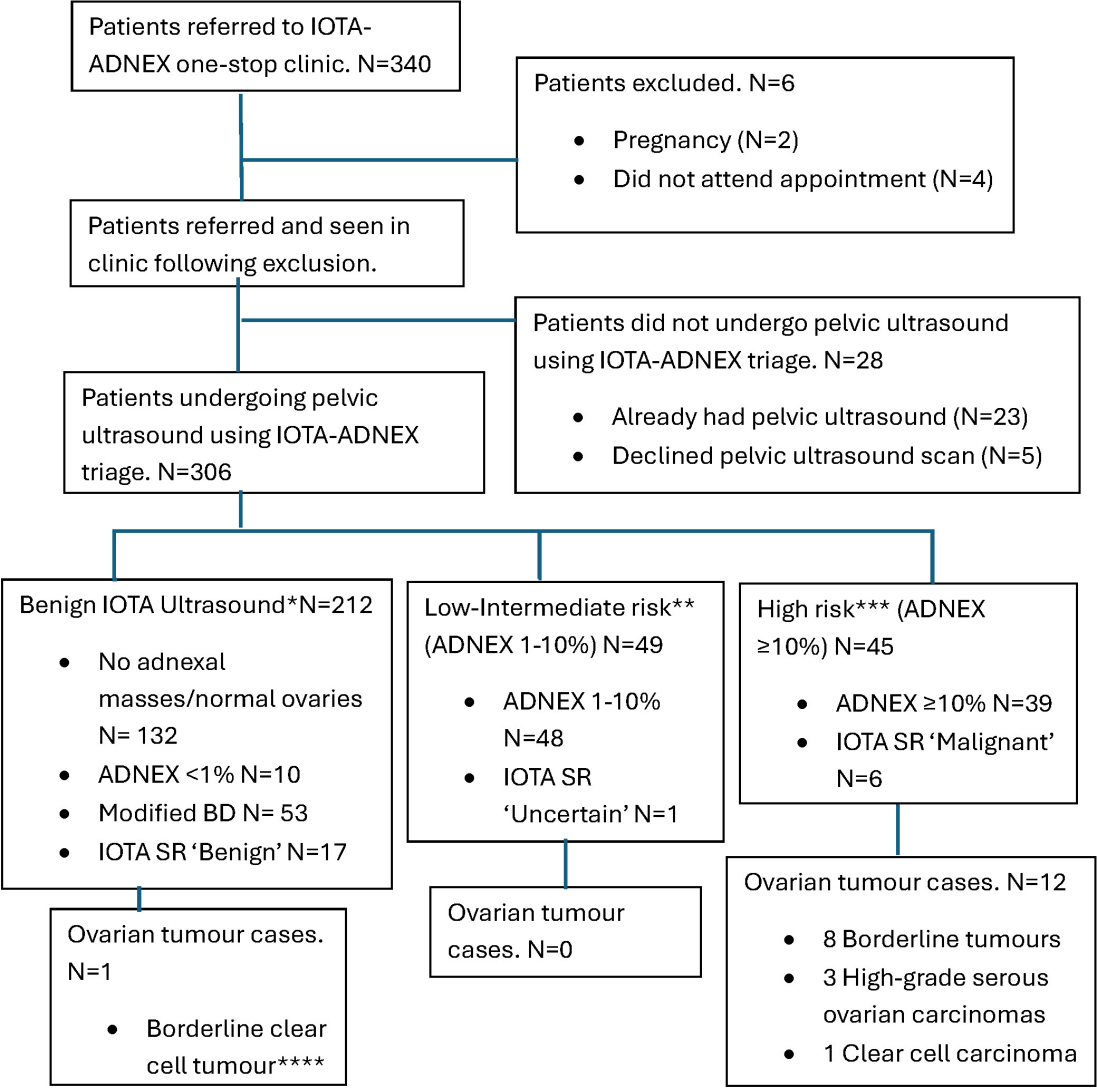
Flowchart of patients included, excluded, undergoing IOTA scans and ovarian tumour outcomes. N (number of patients). *Definition of Benign IOTA Ultrasound: normal ovaries and no adnexal mass found on ultrasound, ADNEX score <1%, IOTA SR ‘Benign’. **Definition of Low-Intermediate risk: ADNEX 1%–10%, IOTA SR ‘Uncertain’. ***Definition of High Risk: ADNEX ≥10%, IOTA SR ‘Malignant’. ****IOTA ultrasound score: 0% as no obvious adnexal masses were seen, but a large fibroid suspicious for sarcomatous changes was noted on ultrasound. Histology confirmed a benign fibroid, but an incidental finding of a borderline clear cell tumour in the ovary. ADNEX, Assessment of Different Neoplasias in the Adnexa; IOTA, International Ovarian Tumour Analysis; SR, Simple Rules.

Over the study period, 340 patients were referred, and 334 patients were seen in the OSC. Six patients were excluded: two patients were pregnant and four did not attend the appointment (figure 2). Patient characteristics are summarised in table 1. 92% (306/334) of patients underwent IOTA ultrasound assessment. Of those who did not undergo IOTA assessment (28/334), most had prior imaging (n=23) and the remaining declined IOTA ultrasound (n=5). 55% (169/306) had previous pelvic ultrasounds before IOTA-ADNEX assessment.

**Table 1:** Patient characteristics: age group, ethnicity and menopausal status

Patient demographics	Total (n=334)
Age	
Under 20	4 (1%)
20–30	20 (6%)
30–40	58 (17%)
40–50	102 (31%)
50–60	56 (17%)
60–70	37 (11%)
70–80	29 (9%)
80+	28 (8%)
Ethnicity	
White British	75 (22%)
British	63 (19%)
Asian/Asian British Pakistani	29 (9%)
Asian/Asian British Indian	45 (13%)
Asian/Asian British Bangladeshi	6 (2%)
Black/Black British Caribbean	19 (6%)
Black/Black British African	10 (3%)
Chinese	4 (1%)
Mixed	5 (1%)
Other	49 (15%)
Not specified	29 (9%)
Menopausal status (based on clinical history)	
Premenopausal	173 (52%)
Perimenopausal	26 (8%)
Postmenopausal	135 (40%)

A total of thirteen histologically confirmed ovarian tumour cases (4%) were diagnosed: 9 borderline tumours (serous borderline n=6, mucinous borderline n=2, clear cell borderline n=1) and four invasive OC (high-grade serous n=3, clear cell carcinoma n=1). All invasive OC was identified following the IOTA-ADNEX scan with no missed invasive OC during this study period. All patients were followed up via electronic hospital records in their presenting hospital for a minimum of 5 months. Additionally, no further invasive OC was identified in centralised Pan Birmingham gynaecological cancer centre MDT meetings that covered both hospitals.

### Benign IOTA findings (n=212, 69%): ovarian tumour cases n=1

Benign IOTA classification was defined as ultrasound reporting normal ovaries or no obvious adnexal masses (n=132), ADNEX score <1% (n=10), modified BD (n=53) or IOTA SR ‘Benign’ (n=17). 7 underwent surgery and had confirmed benign histology, the remaining 7 patients are awaiting surgery under the general gynaecology team.

A borderline ovarian clear cell adenofibroma was identified incidentally on histology following total abdominal hysterectomy and bilateral salpingo-oophorectomy performed for clinical suspicion of sarcomatous changes within a postmenopausal enlarging fibroid, with preoperative IOTA ultrasound and CT demonstrating no adnexal masses.

### Low-intermediate risk group: IOTA-ADNEX <10% (n=49, 16%): ovarian tumour cases n=0

48 patients were found to have an IOTA-ADNEX score of 1%–10% and one patient was deemed ‘Uncertain’ using IOTA SR. No cancer cases were diagnosed, and the majority (6/11) of patients had confirmed benign histology, the rest are pending surgery under the general gynaecology team.

### High risk group: IOTA-ADNEX ≥10% (n=45, 15%), ovarian tumour cases n=12

39 patients had an IOTA-ADNEX score ≥10%, 6 with IOTA SR classification ‘Malignant’. Following surgery, benign histology was found in twelve patients, and two patients are pending surgery (cancer centre n=1, general gynaecology n=1). One patient has undergone surgery with intraoperative appearances suggestive of disseminated OC, and histology is currently pending. This group yielded the highest ovarian tumour detection rate (n=12, 27%). Among patients who underwent surgery with a cancer centre, there were eight borderline ovarian tumours, three high grade serous OC (stages: 3A1ii, radiologically stage 4B and one un-staged). One clear cell carcinoma was diagnosed following surgery with a cancer unit team.

### Surgical outcomes following IOTA-ADNEX compared with RMI at 250-threshold

Surgical outcomes observed following IOTA ultrasound scan and predicted counterfactual outcomes based on retrospectively calculated RMI at 250-thresholds are shown in table 2. Using IOTA-ADNEX at a 10% threshold, 10% (32/334) of patients underwent surgery under the general gynaecology and cancer unit team. In comparison, 30% (94/334) would have undergone surgery under the same teams if management was based on retrospectively calculated RMI using NICE/RCOG thresholds. 62% (8/13) of diagnosed ovarian tumours had a calculated RMI ≥250. Significantly, five ovarian tumours had a predicted RMI score <200; this included two invasive cancers and three borderline tumours. If RMI had been used, the 2 cases of invasive cancers (one clear cell OC, one high grade serous OC) would not have been managed in a cancer centre setting.

**Table 2:** Patients undergoing surgery following IOTA scan and predicted RMI triage to surgery based on 250 threshold

Menopausal status	Observed surgical outcomes following IOTA scanning (n=306)			Total	Predicted triage to surgery in general gynaecology/cancer unit RMI 25–250*	Predicted triage to surgery in cancer centre RMI ≥250*	Total
	General gynae	Cancer unit	Cancer centre				
Premenopausal (including perimenopausal)	16	4	10	30 (9.8%)	51	8	59 (19.3%)
Postmenopausal	10	2	13	25 (8.2%)	43	15	58 (19.0%)
**Total**	26	6	23	55 (18.0%)	94	23	117 (38.2%)

* Predicted outcomes based on retrospectively calculated RMI.

IOTA, International Ovarian Tumour Analysis; RMI, Risk of malignancy index.

### Diagnostic test characteristics

Retrospectively calculated RMI at 250-threshold had a sensitivity of 66.7% (95% CI 39.1% to 86.2%) and specificity of 94.9% (95% CI 91.8% to 96.9%) for OC detection. The positive predictive value (PPV) was 34.8% (95% CI 16.4% to 57.3%) with negative predictive value (NPV) 98.3% (95% CI 96.1% to 99.5%). Should RMI at the 200-threshold be used in the postmenopausal cohort, 22.5% (69/306) patients would have undergone surgery (RMI ≥200 advised laparotomy under cancer centre team n=16, RMI 0–200 requiring surgical intervention with cancer unit/general gynaecology team n=53).

Using the IOTA-ADNEX model at a 10% threshold, the calculated sensitivity was 92.3% (95% CI 66.7% to 98.6%) and specificity 88.7% (95% CI 84.6% to 91.9%). PPV was 26.7% (95% CI 14.6% to 41.9%) with NPV of 99.6% (95% CI 97.9% to 100%).

### Patient process outcomes following IOTA-ADNEX ultrasound

60% (184/306) were discharged within 3 months and this pathway achieved a 42% (139/334) same day discharge rate. Further CT/MRI imaging was performed in 17% (35/212) of patients with benign IOTA ultrasound findings, 31% (15/49) in the low-intermediate risk group and 89% (40/45) in the high-risk group (table 3).

**Table 3:** Patient process outcomes by IOTA-ADNEX score

	Discharge within 3 months of referral	Same day discharge	Further surveillance	Further imaging	
				CT	MRI
Benign IOTA findings (n=212)	165 (78%)	126 (59%)	30 (14%)	17 (8%)	18 (9%)
Low-Intermediate risk (n=49)	16 (33%)	5 (10%)	22 (45%)	0 (0%)	15 (31%)
High risk (n=45)	3 (7%)	0 (0%)	15 (33%)	22 (49%)	18 (40%)

IOTA, International Ovarian Tumour Analysis.

### Qualitative findings

Five themes were identified from the qualitative analysis. A summary table with supporting quotations, practical recommendations for NHS trusts and participant contribution for each theme can be found in online supplemental table 1.

#### Theme 1: organisational infrastructure

##### Facilitators

Prior experience with OSC models for other gynaecological conditions such as postmenopausal bleeding enabled an established operational framework that could be adapted for this clinic.

Dedicated clinical leadership proved essential as initial absence of designated leads created challenges. Once gynaecologist and sonographer lead roles were established, implementation proceeded more smoothly with improved coordination between services. At SWBH, sonographers valued the support of a lead gynaecology sonographer who oversaw quality assurance and developed an IOTA-reporting proforma to guide scan reporting.

##### Barrier

Sonographers reported that suboptimal imaging quality from current scan machines hindered confident interpretation and could necessitate additional investigations. Therefore, higher-specification ultrasound scanning equipment was required for more accurate reporting. Staffing limitations, including the national sonographer shortage and difficulties in retaining IOTA-certified staff, were a recognised restriction on service capacity and skill development as regular IOTA scan exposure was seen as essential for building confidence and expertise. At Walsall, reliance on a single clinician for scanning and assessment limited the number of patients seen and led to cancellations during their absence. Initial underestimation of appointment lengths led to overbooking of patients, causing delays and clinic overrunning.

### Theme 2: clinical decision making

#### Facilitator

IOTA-ADNEX two-step strategy within an OSC setting enhanced clinical decision-making efficiency. It enables confident triage of benign masses and provides detailed risk stratification to facilitate timely diagnostic decisions and management plans within a single appointment.

This process can help reduce delays in delivering appropriate care for both benign and malignant cases. IOTA-ADNEX assessment was perceived to reduce unnecessary CT/MRI requests, resulting in more efficient resource utilisation.

#### Barrier

Despite the positive clinical impacts, changes take time and can come with resistance. Initial concerns were raised by a clinician of using IOTA-ADNEX rather than RMI as mandated by NICE guidelines. Early uncertainty around score calculation and interpretation, particularly before the IOTA-ADNEX calculator became readily available, undermined confidence in clinical decision-making.

### Theme 3: communication and pathway definition

#### Barrier

The importance of communication pre-implementation was echoed by clinic staff (nursing staff and healthcare assistants) who reported insufficient information about this new service. Clinicians expressed uncertainty in interpreting IOTA-ADNEX scores and requested clear guidance on discharge, surveillance, further imaging, surgery and cancer centre referral. Similarly, concerns were raised over the lack of clarity and ownership for those requiring surgery with benign masses and discharged from cancer pathways. Initial implementation saw most patients referred to MDT meetings as a “safety net”, creating workload concerns.

#### Facilitator

Post-clinic pathways standardised management based on IOTA-ADNEX scores were established. Dedicated gynaecology leadership and growing familiarity with the model facilitated appropriate MDT referrals and helped manage the initial rise in MDT workload. Clinician sentiments echoed this concept.

### Theme 4: professional collaboration and training support

#### Barriers

Initial resistance to inter-professional working, particularly regarding sonographers’ expanded diagnostic roles with concerns about quality control and result interpretation, was raised.

#### Facilitators

Although resistance to interprofessional working posed a barrier to professional collaboration, interviews identified instances where professionals embraced working together. Real-time case discussion during clinic fostered positive professional relationships and immediate feedback opportunities, helping “professional development and team development”.

Regular multidisciplinary quality assurance sessions proved valuable for continuous learning and development. These sessions, sometimes including an IOTA specialist adviser, enabled case review, image-histology correlation and continuous skill development.

### Theme 5: patient experience

#### Facilitator

The majority of participants felt that patients benefitted from this model due to reduced waiting times, fewer appointments and same-visit results discussion. Staff perceived patients appeared “happier and more reassured” and “less anxious” following OSC visits.

#### Barrier

While from staff’s perspective the service appears beneficial to patients, some raised that this could also be ‘overwhelming’ for some who were not prepared for the possibility of bad news.

This may be related to insufficient pre-appointment patient information. The fast referral to appointment time and absence of clinic-specific information limited the opportunity for adequate patient preparation, particularly for those with lower health literacy and/or language barriers who may have needed further support.

Infographics summarising the principal findings are presented in online supplemental figure 5.

### Patient satisfaction survey

Between February and June 2025, 70 patients were invited to complete an online patient satisfaction survey (online supplemental figure 3), with 21 responses received. All respondents were either ‘very satisfied’ or ‘satisfied’ with the service and found it either ‘extremely’ or ‘very’ valuable to have the ultrasound, results and consultation in one visit. 67% (14/21) reported that ‘getting results on the same day’ was most helpful, with 24% (5/21) reporting reduced anxiety about awaiting results. 71% (15/21) indicated that a patient information leaflet prior to the appointment would have been helpful.

## Discussion

This mixed-method study offers a comprehensive evaluation of how IOTA-ADNEX implementation can positively impact care delivery and patient outcomes while exploring key barriers and facilitators in real-world NHS practice. No invasive cancers were missed with IOTA-ADNEX implementation within an OSC pathway and clinicians involved showed appreciation of the accelerated diagnostic process. High rates of same-day discharge support NHS Faster Diagnosis Standards.^7^ Cancer testing can cause psychological distress, with negative experiences linked to reduced engagement with care.^23^ Staff believed that IOTA-ADNEX triage within the OSC setting contributed to an improved patient experience and better clinical outcome, while patients expressed satisfaction with the service.

This pilot showed a 20 percentage point reduction in benign procedures in a retrospective comparison with RMI threshold using NICE/RCOG protocols. These findings mirror published validation studies when IOTA protocols were performed by expert ultrasound examiners.^24 25^ Notably, we demonstrated similar benefits with IOTA-certified, non-expert examiners. This corresponds with the ROCkeTS study, a prospective diagnostic accuracy study involving qualified, level II sonographers, which found IOTA-ADNEX at 10% threshold outperformed RMI in sensitivity.^11^ Our results support integrating IOTA-ADNEX into routine clinical practice and reinforce its reproducibility in varied clinical settings (general hospital to specialised cancer centre) and with non-expert ultrasound examiners.

High rates of conservative management in postmenopausal women, consistent with RCOG guidance for monitoring benign ovarian cysts, show the potential for service optimisation.^8^ Long-term studies show that the risk of complications including torsion and malignancy is <1%, reinforcing the safety of conservative management and minimising the need for repeated follow-up.^16^ This approach preserves limited NHS imaging capacity while improving patient experience by potentially reducing unnecessary intervention. Wider use of the IOTA-ADNEX two-step strategy, supported by structured pathways and standardised reporting proformas, can streamline care, reduce CT/MRI requests and ease MDT workload. Inadequate standard pelvic ultrasound reporting led to duplicate scans in secondary care, underscoring the need for consistent triage and reporting protocol. From both a patient and service capacity perspective, a single robust scan is preferable to multiple assessments.

The pre-existing OSC infrastructure for suspected endometrial cancer helped facilitate successful implementation of this pathway. There were established frameworks for appointment scheduling and anticipated resource requirements, while clinical and administrative staff had confidence in the OSC model’s effectiveness from previous use in postmenopausal bleeding clinic. Together, these factors enabled a smoother transition and delivery of a streamlined service.

Identifying staffing needs is one challenge, ensuring sufficient availability of IOTA-certified staff is another. In Walsall, the service was led by an IOTA-certified gynaecologist, which simplified care planning by combining clinician and scanner roles, but their absence meant cancelled clinics. Sustaining and expanding the service requires sonographers to perform regular IOTA-ADNEX scans to build expertise and confidence, yet national and local shortages alongside clinical demand present a challenge in ensuring continuity for skill development. Sonographers emphasised the importance of ongoing training support. Providing protected training time with sustained investment in developing and maintaining a certified workforce is invaluable. Strategic workforce planning should anticipate future demand and ensure adequate scan volumes to preserve confidence and competencies. Regular MDT meetings to discuss cases alongside clinicians and expert scanners were important for learning.

There also needs to be confidence in the images that are produced. Sonographers emphasised that the current scanning equipment could not provide images of high-enough definition to allow them to confidently interpret images shown. Pre-implementation, it would be helpful to engage with stakeholders, including sonographers, to gauge their opinions of the current equipment and anticipate any practical needs.

In this evaluation, clinical and imaging leadership was invaluable and aided in securing a multidisciplinary consensus for the new diagnostic pathway. Establishing these roles during the project meant that there was clinical oversight of the clinic, training support and quality assurance in sonography. Allocation of these leadership roles pre-implementation could facilitate early wider-team engagement with clinicians and clinic staff by providing targeted education on the new pathway practicalities as well as education on evidence base and national guidance. Sustainability requires clinician-led implementation, with designated leads in gynaecology and imaging departments, working together to unify and support a stratified ultrasound pathway.

Developing a patient information leaflet explaining the nature and process of the clinic and potential outcomes would better prepare patients. Information should be available in accessible formats and multiple languages to address cultural and language barriers. However, it is important to strike a balance of clearly explaining the purpose of the clinic visit to test for possible OC, without unduly increasing patient anxiety. This preparation could enable patients to bring in support and reduce the experience potentially being ‘overwhelming’ or when delivering unexpected diagnoses to unprepared patients.

### Strengths and limitations

This study’s strengths include its prospective, mixed-method design, evaluating the IOTA-ADNEX implementation in general hospital and specialised cancer centre settings. It combined quantitative clinical outcomes and qualitative insights on the facilitators and barriers to implementation in routine clinical practice using non-expert IOTA certified ultrasound examiners. The qualitative evaluation involved a range of stakeholders and was led by a clinical research fellow involved in the clinic which offered contextual insight. It was co-conducted and reviewed by an independent quality improvement consultant to reduce researcher bias. The relatively small sample size, geographic restriction to two West Midland hospitals and use of the OSC model may limit generalisability across the broader NHS where infrastructure and clinical pathways may show greater variation. Although this was a service evaluation of a pathway within the local context, qualitative findings can potentially offer useful implementation strategies that can inform healthcare practice in similar settings. These approaches can be adapted to align with the specific needs, resources and pathway available across different NHS trusts. Survey results may offer meaningful patient insight, but they should be interpreted cautiously given the limited number of responses. While cost-consequence analysis was reported in the ROCkeTS study and provided supportive evidence for the use of IOTA-ADNEX implementation, a formal cost-consequence analysis was not undertaken here.^26^ Differences in implementation between the cancer centre (sonographer-led scanning) and cancer unit (consultant-led service) were not directly compared, and therefore the impact of these variations on scalability cannot be determined.

Retrospective calculation of RMI scores was used to compare outcomes against prospective IOTA-ADNEX triage, limiting direct comparability and necessitating future studies with contemporaneous controls. It may overestimate differences in intervention rates due to unmeasured factors including clinical decision-making factors, retrospective application of RMI thresholds, patient preferences, comorbidities and surgical fitness. Sensitivity and specificity estimates may be affected by verification bias as it is possible that rarely patients discharged may later be diagnosed with cancer. During early implementation, the IOTA-SR model was used due to the temporary unavailability of the IOTA-ADNEX calculator and initial limited awareness of the model.

## Conclusion

Implementation of IOTA-ADNEX ultrasound triage within OSC involving IOTA-certified, non-expert ultrasound examiners represents a valuable improvement opportunity in diagnostic pathways. We show that this model can offer improvement in resource capacity, potential reduction in benign surgery and improved patient experience, especially where expert ultrasound examiners may not be available. However, successful implementation of a new diagnostic pathway is an iterative process that requires thoughtful planning around service delivery. This includes establishing clear leadership roles, ensuring access to appropriate ultrasound equipment and providing supportive structures such as defined patient pathways and standardised reporting templates to promote consistency. Ongoing investment in training and effective communication, particularly within the clinical team, is essential to foster strong professional collaboration and sustained adherence.

These findings support wider IOTA implementation across the NHS, though further multi-site research is needed to capture variation across hospital trusts. Implementation is underway across 11 hospital trusts within the East of England Cancer Alliance. A larger mixed-method evaluation, the ADVOCATE (ADNEX implementation for Ovarian Cysts Triaging East of England) study, is in progress to assess implementation outcomes across more varied hospital settings and geographies to further validate these promising results.

## Supplementary material

10.1136/bmjoq-2025-003909 online supplemental figure 1

10.1136/bmjoq-2025-003909 online supplemental figure 2

10.1136/bmjoq-2025-003909 online supplemental figure 3

10.1136/bmjoq-2025-003909 online supplemental figure 4

10.1136/bmjoq-2025-003909 online supplemental figure 5

10.1136/bmjoq-2025-003909 online supplemental table 1

## Data Availability

No data are available.
