# Supplementary figures and images for: Mixed-methods evaluation of the implementation of IOTA-ADNEX ultrasound triage in NHS secondary care ovarian diagnostic one-stop clinics
Source: BMJ Open Qual. 2026 Apr 20;15(2):e003909. doi: 10.1136/bmjoq-2025-003909 (PMC13110681; doi:10.1136/bmjoq-2025-003909)

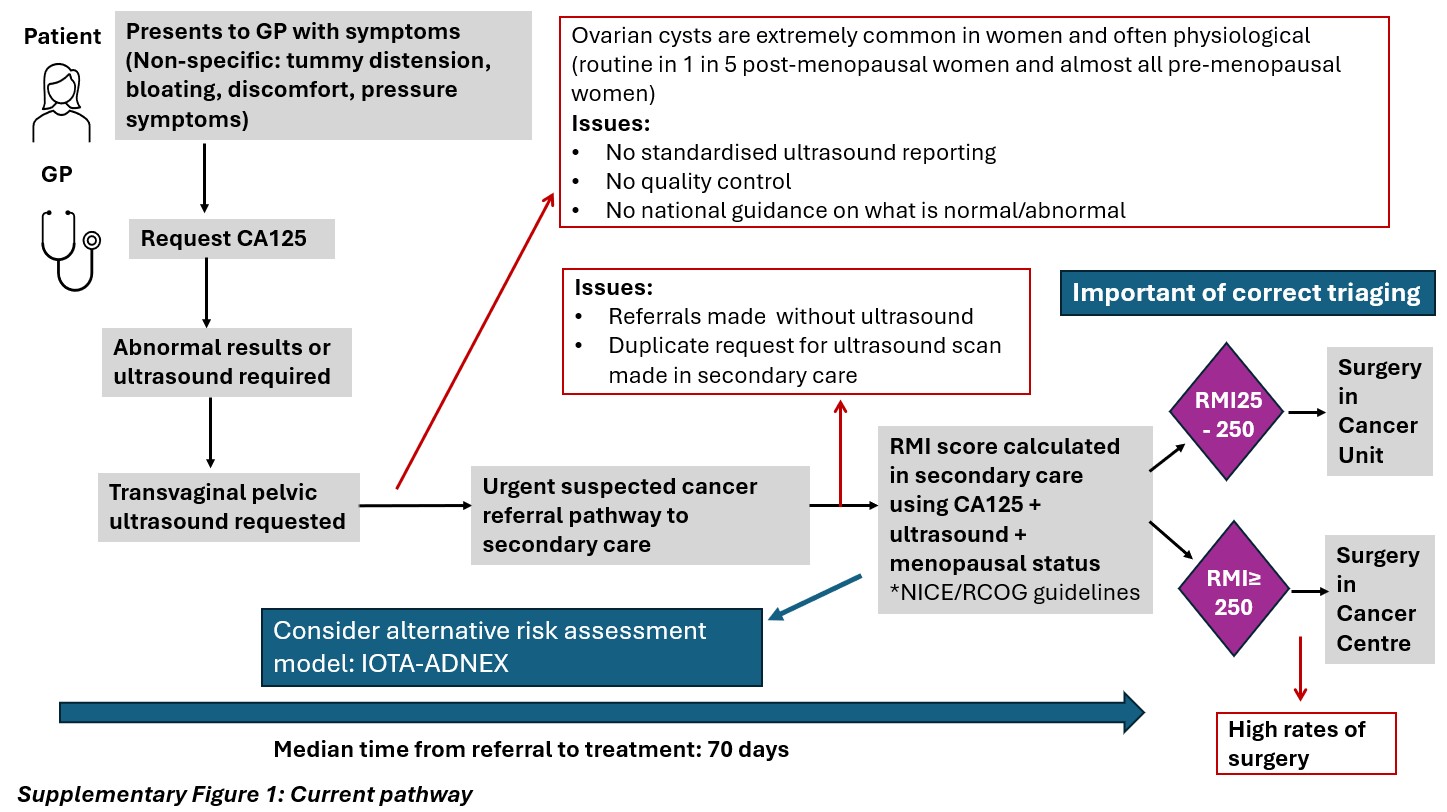

Supplement: online supplemental figure 1 [file bmjoq-15-2-s001.jpeg]

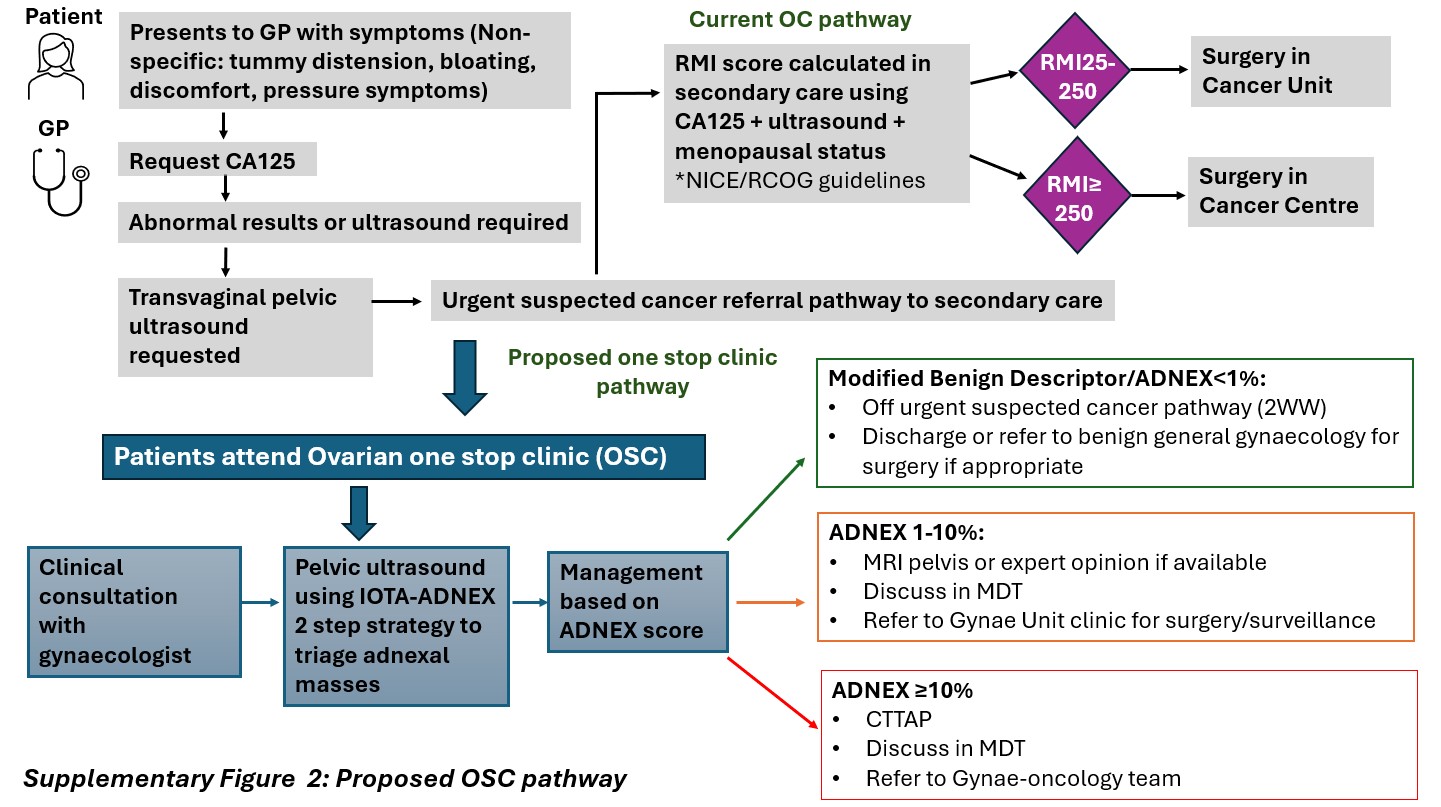

Supplement: online supplemental figure 2 [file bmjoq-15-2-s002.jpeg]

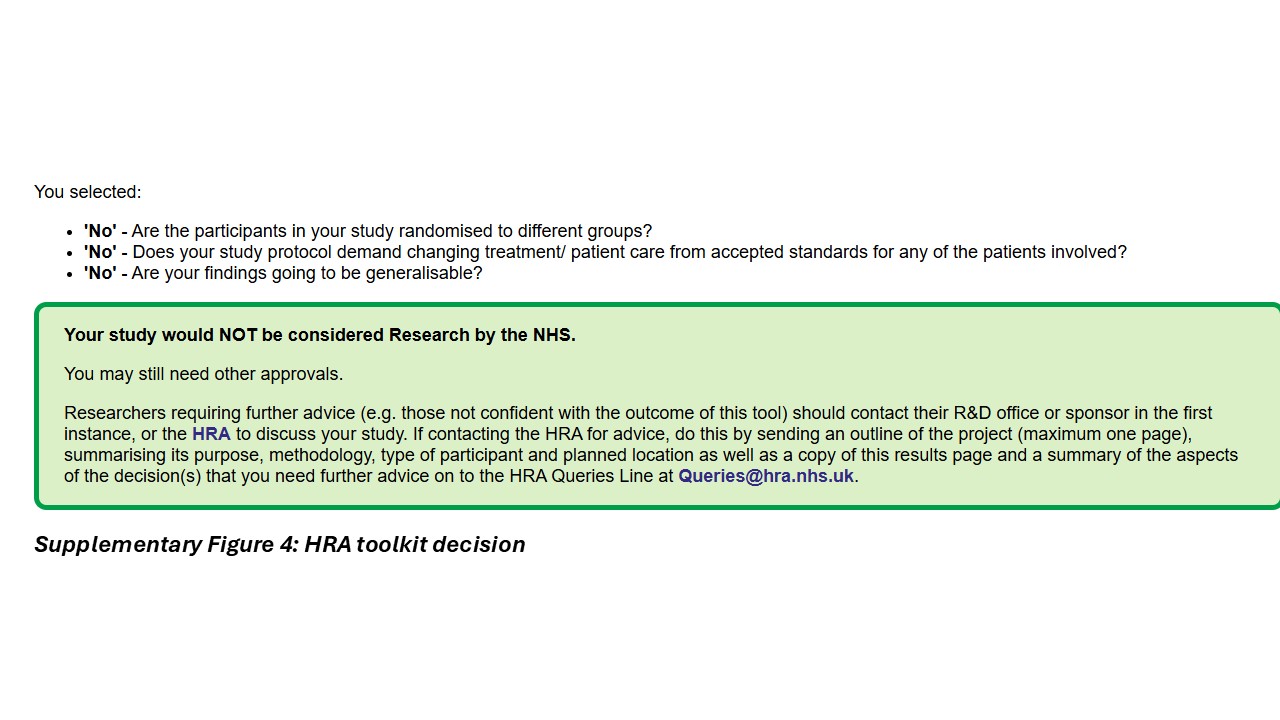

Supplement: online supplemental figure 4 [file bmjoq-15-2-s004.jpeg]
