# Supplementary material for: Mixed-methods evaluation of the implementation of IOTA-ADNEX ultrasound triage in NHS secondary care ovarian diagnostic one-stop clinics
Source: BMJ Open Qual. 2026 Apr 20;15(2):e003909. doi: 10.1136/bmjoq-2025-003909 (PMC13110681; doi:10.1136/bmjoq-2025-003909)
Supplement: online supplemental figure 3 [file bmjoq-15-2-s003.pdf]

Ovarian One Stop Clinic (OSC) - Patient Experience Survey

We aim to provide the very best care and service to our patients. We would like to ask for your help with this by completing the survey below about your experience as a patient with us.

Your views will enable us to improve the quality of care we offer. The survey is voluntary. All information from this survey will be gathered anonymously and treated confidentially. Participation will not affect your on-going treatment in anyway.

Thank you for taking the time to give us your feedback.

* Required

1. **Please input the date of your clinic vist** *

2. **How satisfied were you with the waiting time for your appointment at the Ovarian One Stop Clinic at Birmingham Treatment Centre?** *

- Very satisfied
- Satisfied
- Neither satisfied nor dissatisfied
- Dissatisfied
- Very dissatisfied

3. **Did you understand that you would receive a consultation and an ultrasound scan during the same visit before attending?** *

- Yes, I fully understood
- Yes, I partially understood
- No, I wasn't aware
- Don't know

4. **How valuable did you find having the ultrasound scan, result and consultations in one visit?** *

- ◯ Extremely valuable
- ◯ Very valuable
- ◯ Somewhat valuable
- ◯ Not valuable
- ◯ Don't know

5. **Which aspects of the One Stop Clinic service were most helpful to you?** *

- ◯ Reduced number of hospital visits
- ◯ Getting results on the same day
- ◯ Reduced anxiety about waiting for results
- ◯ Other (Please specify)

6. Please specify the aspect which was most helpful to you? *

[ ]

7. **If you felt able to ask important questions of staff, did you get answers that you could understand?** *

- ◯ Yes, definitely
- ◯ Yes, to some extent
- ◯ No
- ◯ I did not have opportunity to ask
- ◯ I did not feel able to ask questions
- ◯ I did not need to ask questions

8. **Would you have found it helpful to have a patient information leaflet prior to your appointment?** *

- ◯ Yes
- ◯ No
- ◯ Not sure

9. **Thinking about your visit to Ovarian One Stop Clinic, overall, how was your experience of our service?** *

- ◯ Very good
- ◯ Good
- ◯ Neither good nor poor
- ◯ Poor
- ◯ Very poor
- ◯ Don't know

10. **Please can you tell us why you gave your answer?**

11. **Please tell us about anything that we could have done better.**

About you

The following questions will help us to understand how experiences vary between different groups of the population. We will keep your answers completely confidential. Please remember, all the questions should be answered from the point of view of the patient.

You do not have to answer these questions if you prefer not to.

12. **Do you have any of the following physical or mental health conditions, disabilities or illnesses that have lasted or are expected to last 12 months or more? (Select all that apply)**

- ☐ Autism or autism spectrum condition
- ☐ Blindness or partial sight
- ☐ Deafness or hearing loss
- ☐ Dementia or Alzheimer's disease
- ☐ Learning disability
- ☐ Mental health condition
- ☐ Prefer not to say
- ☐ None of the above

13. **What is your sexual orientation?**

- ○ Heterosexual/Straight
- ○ Gay/Lesbian
- ○ Bisexual
- ○ Other
- ○ Prefer not to say

14. **What is your age group?**

- ○ 0 - 15 years
- ○ 16 - 24 years
- ○ 25 - 49 years
- ○ 50 - 74 years
- ○ 75 - 84 years
- ○ 85 years and over

15. **To which of these ethnic groups would you say you belong to?**

- ◯ Asian/Asian British
- ◯ Black African/Black Caribbean/Black British
- ◯ Mixed/Multiple Ethnic Groups
- ◯ White
- ◯ Other
- ◯ Prefer not to say

This content is neither created nor endorsed by Microsoft. The data you submit will be sent to the form owner.

Microsoft Forms
