# Supplementary material for: Mixed-methods evaluation of the implementation of IOTA-ADNEX ultrasound triage in NHS secondary care ovarian diagnostic one-stop clinics
Source: BMJ Open Qual. 2026 Apr 20;15(2):e003909. doi: 10.1136/bmjoq-2025-003909 (PMC13110681; doi:10.1136/bmjoq-2025-003909)
Supplement: online supplemental figure 5 [file bmjoq-15-2-s005.pdf]

Implementation of IOTA-ADNEX ultrasound triage in NHS secondary care ovarian diagnostic one-stop clinics

334 patients with suspected ovarian cancer accessed a new one-stop clinic pathway across 2 NHS Hospitals (Sandwell and West Birmingham & Walsall Manor Hospital) during June 2023-2025, where they received a clinical consultation, pelvic ultrasound scan using the IOTA-ADNEX two-step strategy ultrasound risk assessment model and management plan all in a single visit.

Timely Diagnosis

- This pathway has achieved a 42% same day discharge rate, supporting NHS Faster Diagnosis Standards, without missing any invasive ovarian cancers during the study period.

Reduction in benign surgical rates

- 20-percentage point reduction in benign surgical rates with ADNEX model, compared to retrospectively calculated RMI. This can enable streamlining of gynaecological services and improve surgical capacity.

Key Implementation Facilitators

- Designated gynaecological and imaging leads
- Timely decision-making capabilities
- Standardised reporting proformas
- Regular multidisciplinary quality assurance session

Implementation Requirements

- Comprehensive pre-implementation team education and engagement
- Protected training for sonographer skill development
- Standardised post-clinic patient pathways

To learn more contact: Dr Vivian Do: v.n.d.do@bham.ac.uk

BMJ Open Quality paper in revision

UNIVERSITY OF BIRMINGHAM

Walsall Hospitals NHS Trust

Sandwell and West Birmingham Hospitals NHS Trust

modality partnership A Commitment to Care

Funded by NHS Cancer Programme

SBRI HEALTHCARE
