# Supplementary material for: Mixed-methods evaluation of the implementation of IOTA-ADNEX ultrasound triage in NHS secondary care ovarian diagnostic one-stop clinics
Source: BMJ Open Qual. 2026 Apr 20;15(2):e003909. doi: 10.1136/bmjoq-2025-003909 (PMC13110681; doi:10.1136/bmjoq-2025-003909)
Supplement: online supplemental table 1 [file bmjoq-15-2-s006.pdf]

Supplementary Table 1: Summary of themes, supporting quotations, number of participants contributing to each theme and practical recommendations for NHS Trusts

	**Supporting Quotation**	**Practical Recommendations for NHS Trusts**
**Theme 1: Organisational infrastructure**<br>Participants contributing to theme: 8		
Facilitators<br>Previous use of a one stop clinic experience	*'[...] already had a well-established one stop clinic for postmenopausal bleeding. The one stop clinic is an advance on previous ways of working.'* Gynaecologist 1<br>*'We had familiarity with the one stop clinic approach due to other one stop clinics. Now there are 5 patients per clinic, like with PMB'* Administrative staff 1<br>*'The one-stop model is well established in gynaecology for other conditions such as post-menopausal bleeding to manage the patient with one hospital attendance. Use of one-stop with the IOTA scan is a very progressive way to manage ovarian cancer.'* Gynaecologist 2	Leverage existing infrastructure<br>• Build on established OSC models for other conditions for smoother implementation<br>Appoint dedicated leadership<br>• Clinical leadership can ensure adherence to IOTA models/patient pathways<br>• Imaging leadership can help maintain image quality, consistency and provide ongoing training<br>Invest in quality equipment<br>• Ensure high-specification ultrasound equipment available before implementation to enable adequate image quality/ confident image assessment<br>Address workforce planning<br>• Account for shortages by securing sonographer time, consider recruitment/retention strategies, ensure ongoing support/training<br>Ensure ADNEX calculator availability<br>• Available via the Gynaia website for IOTA-registered members.<br>• Ensure individual IOTA memberships for relevant/involved staff
Dedicated gynaecology and gynae-sonographer leadership	*'There were initial problems around the clinicians and in identifying a named gynae consultant lead for the clinic. The lead is now [...] and things are working well.'* Gynaecologist 1<br>*'It helps now that [...] is now Gynae lead for scanning'* Sonographer 2<br>*'There was no 'hands on' leadership which is needed for this type of change'* Gynae-oncologist 1	
Barriers<br>Lack of high specification ultrasound equipment	*'[...] a lot of challenges on the imaging side as the equipment is not really good enough for the quality of scans we need. It's a massive block on how the imaging can be done.'* Sonographer 1<br>*'There has to be good enough equipment for the ultrasounds...we need better quality scans than we currently have'* Gynaecologist 1	
Staffing limitations including sonographer shortages	*'Securing sonographers for the clinic was difficult, sonographers were already allocated to scanning sessions and short staffing with gaps in staffing and sonographers leaving was a problem'* Administrative staff 1<br>*'There is a national shortage of sonographers'* Gynaecologist 1<br>*'The limiting factors is having a gynae-consultant who can do the scan or it requires a sonographer who has an interest in pelvic scanning'* Gynaecologist 3<br>*'Years ago there was a general gynae one stop clinic. It felt like it was very effective, but due to missing resources like not enough sonographers, the services went. I can anticipate this could happen again.'* Clinic staff 2	
**Theme 2: Clinical decision making**<br>Participants contributing to theme: 8		

Facilitators<br>Staff perception that OSC setting with IOTA-ADNEX 2 step strategy enabled timely decision making	*'It saves a lot of time in the decision making…It [IOTA-ADNEX in the OSC] facilitates care planning, we can take the decision then and there.'* Gynae-oncologist 3<br>*'The clinic gives the confidence to make the care plans, and it is a lot quicker.'* Gynaecologist 3<br>*'We are getting 2 week wait patients in sooner with the one stop clinic option being available. It has allowed for quicker discharged and helped with targets.'* Administrative staff 1	Active clinician engagement pre-implementation<br>• Provide education and awareness of robust evidence for IOTA-ADNEX prior to implementation<br>• Communicate evidence base/recommendation in BGCS Ovarian Cancer guidelines and rationale for IOTA-ADNEX implementation(13)
Staff perception in the possible reduction of further imaging requests	*'When we get it right [a clear image which facilitates accurate reporting] far fewer CT and MRI scans are needed – it's better for patients and for the hospital'* Sonographer 1<br>*'…better use of CT scans as and when these are clinically needed.'* Gynaecologist 4	
Barriers Resistance to working outside NICE guidelines	*'As this stands, NICE rejects this technology – why is it being introduced? This needs to be addressed head on in any trial if the trial means going against current NICE guidance.'* Gynae-oncologist 1<br>*'[…] some gynaecologists who are very change resistant'* Sonographer 2	
**Theme 3: Communication and pathway definition**<br>Participants contributing to theme: 8		
Barriers<br>Lack of communication of the new diagnostic pathway for staff members	*'We weren't sure how the clinic was meant to be run initially'* Clinic staff 1<br>*'[…] no specific briefing for this clinic. This was not much of a difference to other 2 week wait clinics, it is the scanning that is quite specific and makes the difference.'* Gynae-oncologist 3	Clear communication with all staff<br>• Comprehensive briefing for all team members (clinical, administrative and clinic support staff) about the new pathway and rationale before implementation<br>Develop clear care pathways<br>• Create standardised flowcharts linking ADNEX scores to the next management steps to guide clinical decision making<br>• A pathway can provide clarity on how to interpret ADNEX risk scores and when further imaging is indicated to increase confidence in model use, ensuring appropriate MDT referrals<br>• Create standardised reporting: Implementing ADNEX reporting proforma to ensure consistent IOTA terminology across service
Lack of clear pathways following ADNEX scan	*'The patient pathway beyond the OSC needs to be developed. With a clear flowchart of the standardised next steps linked to the ADNEX score'* Gynaecologist 2<br>*'How to interpret the numbers? … Clinicians need clarity'* Gynae-oncologist 1	
Surge in MDT referrals	*'The MDT was being used as a safety net, but we realise that there was no need for all these cases to come to the MDT once the clinic is established'* Gynae-oncologist 2	
Facilitators Clinical perception of possible reduction in MDT referrals	*'If it is working as intended, it should be a better use of resources. But if the vast majority come through to the MDT anyway, it's just an extra layer. But if it's effectively triaging patients out, it's well worth it.'* Gynaecologist 4	
**Theme 4: Professional Collaboration and Training Support**		

Participants contributing to theme: 4		
Barrier Resistance to interprofessional working	*'The Sonography quality control is just not there.'* Gynae-oncologist 1	Facilitate regular quality assurance sessions<br>• Schedule routine MDT meetings between sonographers and clinicians to review cases and correlate images/interpretation with histological diagnoses to improve skills and confidence<br>• Include gynaecological ultrasound specialist in quality assurance meetings to provide insightful feedback/advice on image quality and interpretation<br>Provide ongoing training<br>• Invest in continuous education on IOTA-ADNEX model
Facilitators Trust between clinical professionals	*'The chat with the sonographers to share ideas and interpret the scans is really helpful'*<br>*'[…] we are building trust and confidence. It is helping professional development and team development.'*<br>Gynae-oncologist 3	
Support for quality assurance sessions	*'[…] scheduled MDT meetings to review how the clinic is working and reviewing the cases together are really important.'* Sonographer 1	
**Theme 5: Patient Information and Experience**<br>Participants contributing to theme: 10		
Facilitator Staff perceived beneficial patient experience	*'It's good that patients have an ultrasound and can have their questions answered afterwords. I feel that patients come out happier and more re-assured'* Clinic staff 1<br>*'We notice patients are less anxious after'* Clinic staff 3<br>*'There is a reduction in patient anxiety'* Gynaecologist 4<br>*'They are getting a good service with quicker decision on diagnosis for most patients'* Gynaecologist 1	Development of patient information leaflets<br>• Create and distribute patient information leaflet explaining the one-stop clinic process, pelvic ultrasound, what to expect and possible outcomes. Give to patients prior to appointment<br>• Ensure appointment letters explain the purpose of the clinic sensitively<br>• Tailor information delivery to varying levels of health literacy and socioeconomic backgrounds
Barriers Staff perceived potential downsides for patients	*'It can be overwhelming for women from the populations we are dealing with, their socioeconomic stata and how much they can take from the information'* Gynaecologist 2<br>*"It is sometimes a challenge as patients come unprepared for bad news if it is a cancer diagnosis, […] . It can be overwhelming for the patient'* Gynae-oncologist 3	
Lack of information for patients prior to clinic appointment	*'Some patients think they are just coming for a chat…they need sufficient information'* Gynaecologist 2<br>*'Often patients are unsure why they are attending the clinic, but this also happens in other clinics'* Clinic staff 4	
